# Which factors influence treatment decision in fragility fractures of the pelvis? - results of a prospective study

**DOI:** 10.1186/s12891-021-04573-2

**Published:** 2021-08-13

**Authors:** Ludwig Oberkircher, Julia Lenz, Benjamin Bücking, Daphne Eschbach, René Aigner, Christopher Bliemel, Carsten Schoeneberg, Steffen Ruchholtz, Juliana Hack

**Affiliations:** 1 Center for Orthopaedics and Trauma Surgery, Philipps University of Marburg, University Hospital Giessen and Marburg GmbH, Marburg, Germany; 2 Department of Orthopaedics, Trauma Surgery and Geriatric Trauma, DRK-Hospital Nordhessen, Kassel, Germany; 3 Department of Orthopedic and Emergency Surgery, Alfried Krupp Hospital, Essen, Germany

**Keywords:** Pelvis, Fracture, Fragility fracture, Osteoporosis, Treatment, Sacral fracture

## Abstract

**Background:**

The aim of the present study was to describe specific characteristics of patients suffering from pelvic fragility fractures and evaluate factors that might influence treatment decisions which may optimize treatment pathways and patient mobility in the future.

**Methods:**

A prospective study with patients suffering from fractures of the pelvis and aged 60 years or above was performed between 2012 and 2016. Data acquisition took place at admission, every day during hospitalization and at discharge.

**Results:**

One hundred thirty-four patients (mean age of 79.93 (± 7.67) years), predominantly female (84%), were included. Eighty-six patients were treated non-operatively. Forty-eight patients underwent a surgical procedure. The main fracture types were B2 fractures (52.24%) and FFP IIb fractures (39.55%). At the time of discharge, pain level (NRS) could be significantly reduced (*p* <  0.001). Patients who underwent a surgical procedure had a significantly higher pain level on day three and four compared to the non-operative group (*p* = 0.032 and *p* = 0.023, respectively). Significant differences were found in the mobility level: patients treated operatively on day four or later were not able to stand or walk on day three as compared to non-operatively treated patients. Regarding B2 fractures, a significantly higher mobility level difference between time of admission and discharge was found in patients treated with a surgical procedure compared to patients treated non-operatively (*p* = 0.035).

**Conclusions:**

Fracture type, mobility level and pain level influence the decision to proceed with surgical treatment. Especially patients suffering from B2 fractures benefitted in terms of mobility level at discharge when treated operatively.

**Level of evidence:**

II

## Background

The incidence of fragility fractures of the pelvis in the elderly is greatly increasing due to rising life expectancy and an aging population [1–5]. A significant correlation with the female sex and advancing age has been found in several studies [6]. The peak incidence of osteoporotic pelvic fractures seems to be in the ninth decade of life [7], and 75% of patients aged 60 or older with pelvic fractures were women in a recently published analysis by Rollmann et al. [8] in accordance with the higher incidence of osteoporosis in women [9, 10].

Due to osteoporosis and increasing ossification as well as rigidity of the ligamentous structures, fragility fractures of the pelvis differ substantially from pelvic fractures of younger patients [6]. The usually used classification of the Association for Osteosynthesis / Orthopaedic Trauma Association (AO/OTA) developed by M. Tile [11] does not apply to the morphologies of pelvic fragility fractures in the elderly and may not correctly reflect the true morphology of such injuries [6]. To give consideration to the particularities of fragility fractures and sacral insufficiency fractures, Rommens and Hofmann developed a new classification system (FFP [fragility fractures of the pelvis] I-IV) [12, 13]. This system can aid in deciding whether an operation is warranted, but the classification has not been validated by any clinical studies to date. Furthermore, as yet there exist no prospective clinical studies to determine further factors influencing treatment decisions regarding fragility fractures of the pelvis. Standardized treatment protocols are still lacking despite the dramatically increasing incidence as well as the increased mortality, reduced mobility and significant loss of social independence in affected patients [6, 9, 14, 15].

The aim of the present prospective study was to evaluate factors that might influence treatment decisions regarding fragility fractures of the pelvis in order to optimize treatment pathways and patient mobility.

## Methods

### Patient recruitment

In our university hospital, we performed a prospective study with patients suffering from fractures of the pelvis between June 1, 2012, and December 31, 2016 (level of evidence: II). The age of the included patients had to be 60 years or above. Furthermore, only patients who were admitted to hospital as inpatients were included in the study. Exclusion criteria were isolated acetabular fractures, high-energy trauma (ISS ≥ 16) and malignancy-related fractures (e.g., osseous metastases). The study was carried out in accordance with the Declaration of Helsinki. Institutional review board (IRB) approval was obtained by the local ethics committee (Ethikkommision Fachbereich Medizin, Philipps Universität Marburg, AZ 22/12). At the time of admission all patients or their legal agents gave their written informed consent for study participation.

### Data acquisition

Data acquisition took place at hospital admission, every day during hospitalization and at discharge from the hospital. All patients underwent initial computed tomography (CT) diagnostics to verify the precise fracture type. At the time of admission the following data were prospectively collected: socio-demographic data (e.g., age and gender), ASA classification (American Society of Anesthesiologists), type of fracture, Mini-Mental Status Test (MMST) and mobility. Mobility was measured by a subclassification in the Barthel index [16]: walking 50 m freely or with crutches (15 points), walking 50 m with a walking frame (10 points), moving only at home with personal help or crutches (5 points) or immobile (0 points). Pelvic fractures were classified using the AO/OTA classification as well as the FFP classification system of Rommens and Hofmann [12]. Patient pain at admission was measured using the Numeric Rating Scale (NRS; from 0, indicating no pain at all, to 10, indicating intolerable pain). When patients showed advanced or severe dementia (MMST < 18), pain was measured using psychometric evaluation of the Pain Assessment in Advanced Dementia (PAINAD) scale (from 0, indicating no pain at all, to 10, indicating intolerable pain) [17]. Data on pain level before fracture and mobility before fracture were collected retrospectively by asking the patient. During hospitalization the following data were collected: type of treatment (non-operative or operative), day of operation after admission (if operative treatment was performed), type of operative treatment (if operative treatment was performed), level of pain using NRS/PAINAD every day and complications (local as well as systemic complications). Furthermore, state of mobility was evaluated every day (ability to stand in front of patient bed, ability to walk inside patient room). At the time of discharge the following data were collected: mobility and level of pain (NRS/PAINAD).

### Pain medication and operative techniques

Pain medication management was conducted according to the in-house analgesic scheme, which is derived from the WHO analgesic ladder but skips mild opioids (first step: nonopioid analgesics such as metamizole, acetaminophen and/or nonsteroidal anti-inflammatory drugs, considering commodities of the patients; second step: strong opioids plus nonopioid analgesics plus adjuvant if applicable). Normally, pain medication started with the first step and then, if that was not sufficient, moved to the second step, but in the case of severe pain, pain medication could be started directly with the second step.

Iliosacral screw was done minimal invasive in prone position under fluoroscopic control. All canulated screws (7,5 mm, aap Biomaterials GmbH, Berlin, Germany) were cemented (Kyphon HV-R Bone Cement, Medtronic, Minneapolis, MN, USA).

For internal fixation of the anterior pelvic ring a minimal invasive, submuscular technique was used. Two pedicle screws are placed supraacetabulary via a bilateral minimally invasive retroperitoneal approach. Soft tissue preparation is subsequently performed subvascular along the os pubis to the opposite side and a pre-curved rod is passed through and attached to the two screws (Marquardt Medizintechnik GmbH, Spaichingen, Germany) [18].

### Statistics

Data were collected in a FileMaker database (FileMaker® Inc., Santa Clara, CA, USA). Statistical analysis was done using SPSS 25 (Statistical Package for the Social Sciences Version 25, IBM Corp., Armok, NY, USA). For the descriptive statistics the means and standard deviation were determined. Values were tested regarding normality distribution using the Kolmogorov–Smirnov test. Normally distributed values were analyzed using Student’s t-test; not normally distributed values were analyzed using the Mann–Whitney U test. Cross-tabulation was analyzed using the Fisher–Yates test and regression coefficient (B). Significance was determined at *p* ≤ 0.05.

## Results

**Fig. 1:**
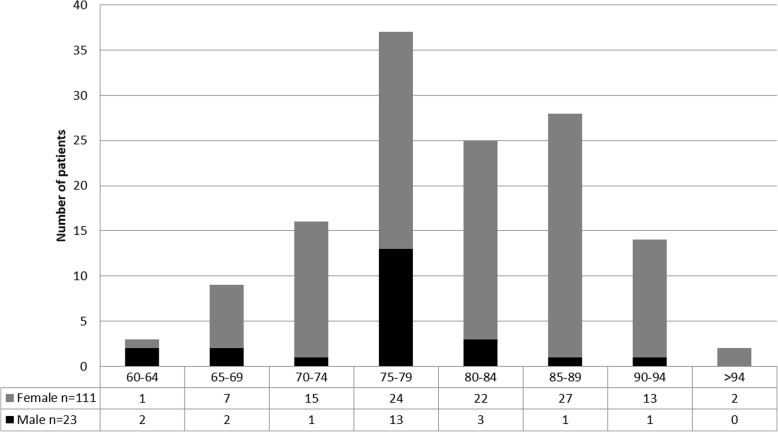
Age and gender distribution of all patients (*n* = 134)

In total, 134 patients were included in the study. Eighty-two of these patients were female (84%), and 23 were male (17.16%). The average age of all patients was 79.93 (± 7.67) years, with average age in the female and male patient groups being 80.77 (± 7.59) and 75.83 (± 6.81) years, respectively. Age and gender distribution is shown in Fig. 1. Osteoporosis had been diagnosed before admission in 41 patients (30.6%).

### Treatment

#### Non-operative treatment

Eighty-six patients (64.18%) were treated non-operatively. Non-operative treatment included analgesia, mobilization under full weight bearing and physiotherapy.

#### Operative treatment

Forty-eight patients (35.82%) underwent a surgical procedure. Operative treatment was done on average on day four after admission (3.88 ± 2.76). Thirty-eight patients (79.17%) were treated with an iliosacral screw, while additional internal fixation was done in 13 patients (27.08%) to address anterior pelvic ring fractures. Spinopelvic fixation was done in five patients (10.42%). Five patients (10.42%) were treated with plate fixation of the posterior pelvic ring.

### Duration of the hospital stay

In total patients were hospitalized 11.57 (±7.17) days on average. Patients treated non-operatively stayed an average of 8.87 (±5.28) days; patients treated operatively had an average hospital stay of 16.29 (±7.64) days (see Table 1).

**Table 1:** Characteristics of the operative and conservative group (all fracture types) and differences between the groups. Values are presented in Mean, Standard Deviation (SD), Median and Interquartile Range (IQR); n = number of patients evaluated, if different from total number; NRS = Numeric Rating Scale, PAINAD = Pain Assessment in Advanced Dementia

All fracture types (***n*** = 134)	Operative group (***n*** = 48) Mean (SD), *Median [IQR]*	Non-operative group (***n*** = 86) Mean (SD), *Median [IQR]*	***p***-value
Age	78.54 (±7)	80.7 (± 7.59)	0.264
Duration hospital stay (days)	16.29 (±7.64)	8.87 (±5.28)	**< 0.001**
Local complications (patients)	7	0	**< 0.001**
Systemic complications (patients)	14	7	**0.001**
NRS/PAINAD at admission	7.57 (± 2.1), *8 [3]*	7.84 (± 1.8), *8 [3]*	0.489
NRS/PAINAD at discharge^a^	3.77 (± 2.4), *4 [3]* (*n* = 42)	4.49 (± 2.2), *5 [3]* (*n* = 57)	0.322
NRS/PAINAD at day three^b^	4.69 (± 2.6), *5 [2]* (*n* = 16)	3.26 (± 2.21), *3 [4]* (n = 57)	**0.032**
NRS/PAINAD at day four^b^	4.83 (± 2.17), *4 [3]* (*n* = 12)	3.19 (± 2.22), *2 [3]* (*n* = 53)	**0.023**
Mobility before trauma	14.06 (± 2.45), *15 [0]*	13.08 (± 2.78), *15 [5]*	**0.017**
Mobility at admission	1.35 (± 3.53), *0 [0]*	2.5 (± 4.04), *0 [5]*	**0.038**
Standing possible (day)^b^	5.52 (±5.01), *4 [5]* (*n* = 21)	2.27 (±2.02), *1 [2]* (*n* = 75)	**< 0.001**
Walking in patient room possible (day)^b^	7 (±4.95), *5 [5.5]* (*n* = 14)	2.72 (±2.62), *2 [2]* (n = 53)	**< 0.001**
Mobility at discharge^a^	6.16 (±4.86), *5 [10]* (*n* = 43)	5.93 (± 5.13), *5 [10]* (*n* = 70)	0.73

^a^One patient died in the operative group, three patients died in the Non-operative group

^b^Patients who were operated before day four were excluded from this examination

### ASA classification

Regarding ASA classification, two patients were classified as ASA I (1.49%), 41 patients as ASA II (30.6%), 79 patients as ASA III (58.96%) and three patients as ASA IV (6.72%). Three patients were classified as ASA V (2.24%).

ASA classification had no significant influence on treatment decision (*p* = 0.426).

### Fracture classification

#### AO/OTA

**Fig. 2:**
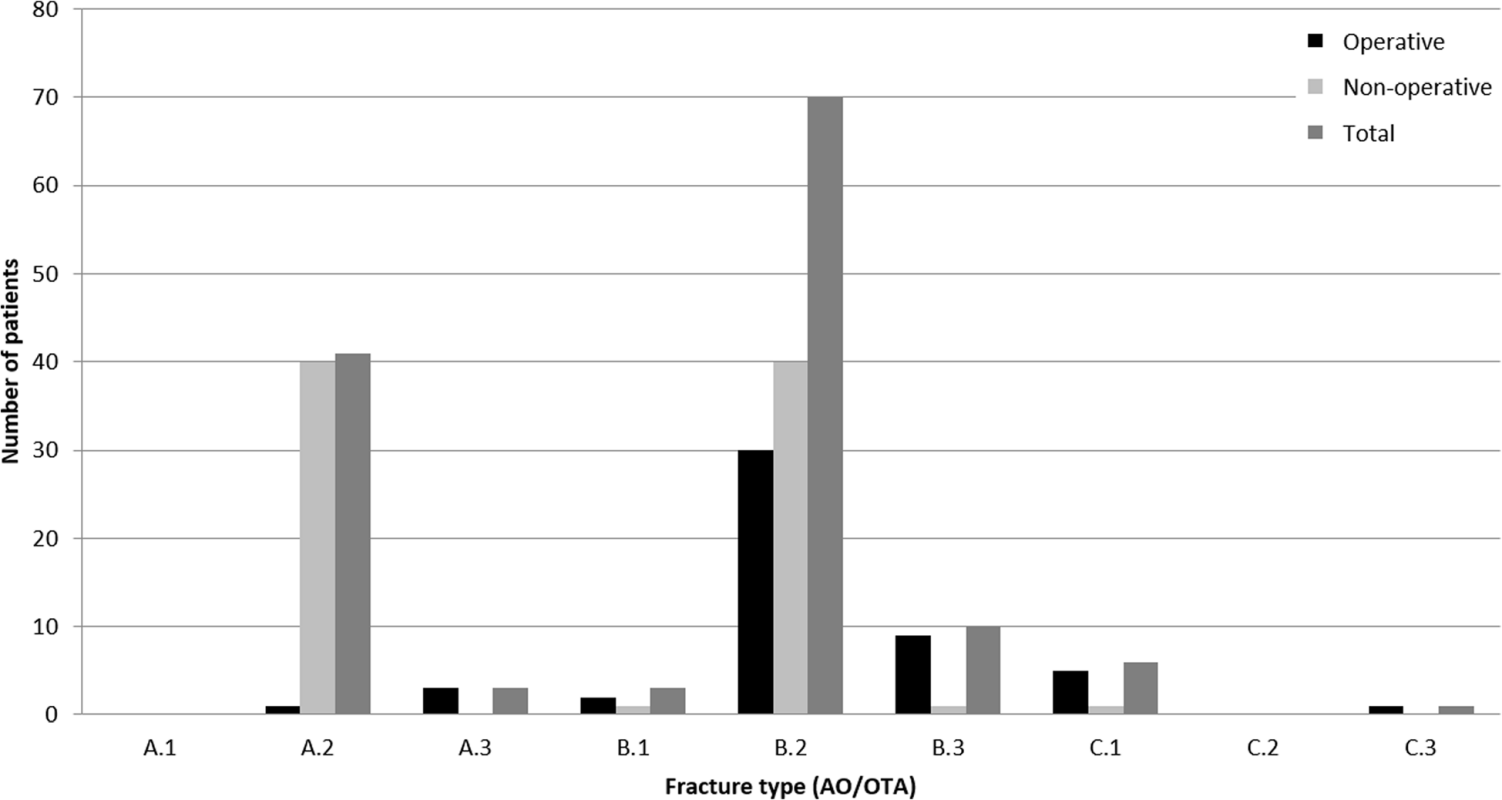
Distribution of fracture types regarding AO/OTA classification (total, operative and non-operative treatment)

Distribution of fracture types regarding AO/OTA classification and respective treatments are shown in Fig. 2.

Most patients suffered from B2 fractures (*n* = 70, 52.24%), whereas only six patients had a C1 fracture (4.48%) and one patient a C3 fracture (0.75%).

Non-operative group.

Forty patients with A2 fractures (97.56%) and 40 patients with B2 fractures (57.14%) were treated non-operatively. Further treatments are shown in Fig. 2.

##### Operative group

Thirty patients with B2 fractures (42.86%) underwent a surgical procedure. Almost all patients with a C1 fracture (*n* = 4, 83.33%) and the patient with a C3 fracture (100%) underwent operative treatment. Further treatments are shown in Fig. 2.

#### FFP (Rommens and Hofmann) classification

**Fig. 3:**
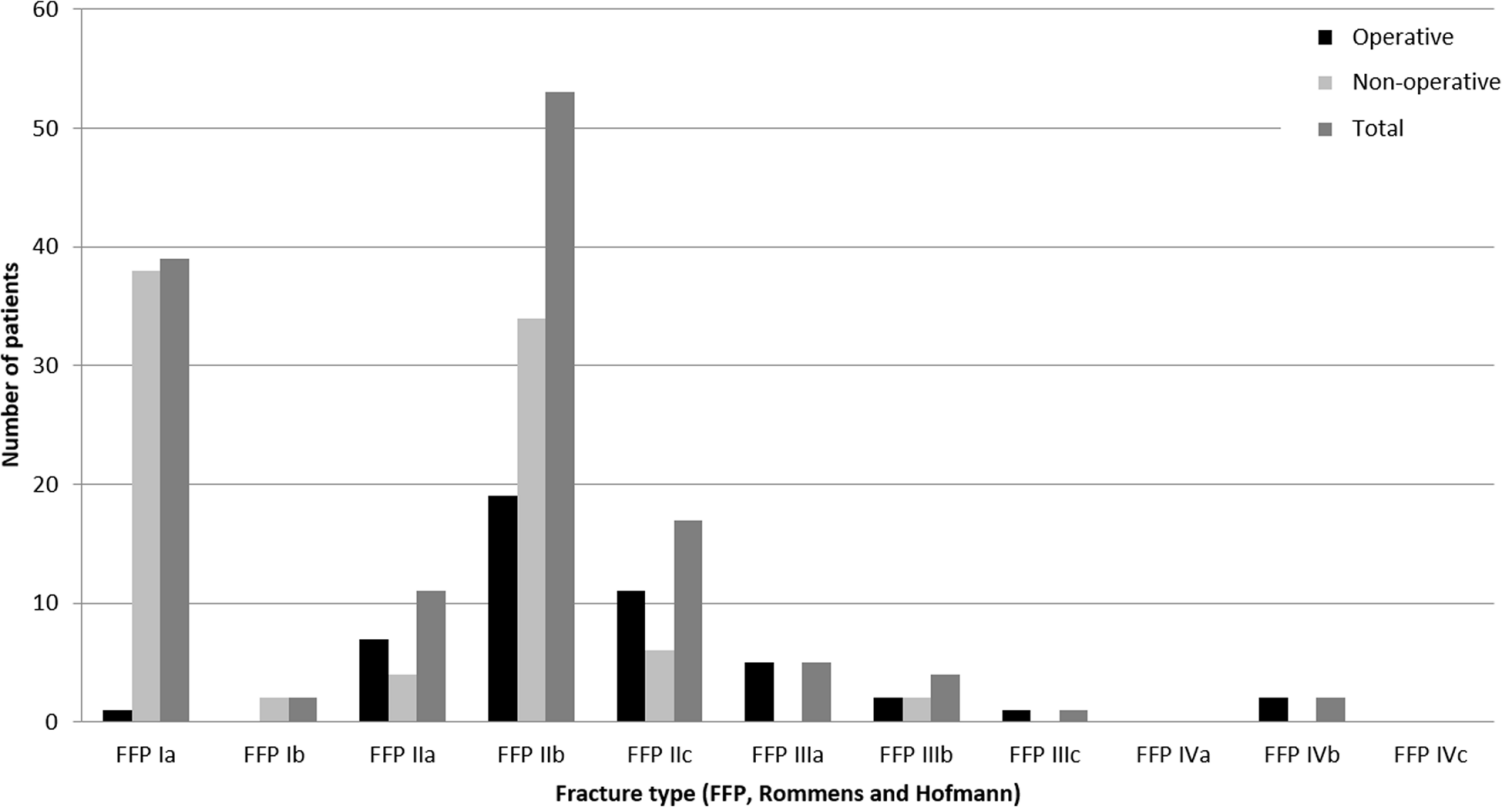
Distribution of fracture types regarding FFP (Rommens and Hofmann) classification (total, operative and non-operative treatment)

Distribution of fracture types according to FFP (Rommens and Hofmann) classification and respective treatments are shown in Fig. 3. Most of the patients suffered from FFP IIb fractures (*n* = 53, 39.55%).

##### Non-operative group

Thirty-eight patients with FFP Ia fractures (97.44%) were treated non-operatively. Thirty-four patients suffering from FFP IIb fractures (64.15%) had non-operative treatment. Further treatments are shown in Fig. 3.

##### Operative group

Nineteen patients who suffered from FFP IIb fractures (35.85%) underwent a surgical procedure. Further treatments are shown in Fig. 3.

#### Evaluation of treatment decision regarding fracture classification

With increasing instability of the fracture type according to AO/OTA as well as Rommens and Hofmann, the probability of an operative treatment increases regarding regression analysis (see Table 2).

**Table 2:** Regression Analysis for fracture classification. Nagelkerke R^2^ regarding AO/OTA = 0.388. Nagelkerke R^2^ regarding Rommens and Hofmann = 0.357. CI = confidence interval. FFP IV was not analyzed due to the small number of cases

Classification	B (regression coefficient)	Odds Ratio (OR)	95% CI of OR	*p*-value
AO/OTA				
A/B	3.74	41.98	[5.5;319.2]	< 0.0005
A/C	5.55	258.0	[14.2;4690.9]	< 0.0005
C/B	−1.82	0.16	[0.02;1.41]	0.1
FFP				
FFP I / II	3.52	33.64	[4.4;256.6]	0.001
FFP I / III	5.08	160.0	[12.9;1983.9]	< 0.0005
FFP III / II	−1.56	0.21	[0.04;1.05]	0.06

#### Complications

In total, complications occurred in 24 patients (17.91%). Four patients died during hospital stay (2.99%). In total three patients died in the non-operative group (3.49%). In the operative group one patient died due to organ failure (2.08%). Complication details are shown in Table 3.

**Table 3:** Distribution of local and systemic complications during hospitalization

**Complications**	All patients (*n* = 134)	Non-operative group (*n* = 86)	Operative group (*n* = 48)
Local:			
Hematoma	4 (2.99%)	0 (0%)	4 (8.33%)
Postop. bleeding	1 (0.74%)	0 (0%)	1 (2.08%)
Wound infection	2 (1.49%)	0 (0%)	2 (4.17%)
Systemic:			
Pneumonia	2 (1.49%)	1 (1.16%)	1 (2.08%)
Urinary infection	16 (11.94%)	6 (9.98%)	10 (20.83%)
Myocardial infarction	2 (1.49%)	1 (1.16%)	1 (2.08%)
Pleural effusion	2 (1.49%)	0 (0%)	2 (4.17%)
Pulmonary embolism	1 (0.74%)	0 (0%)	1 (2.08%)
multiple organ failure	3 (2.24%)	2 (2.33%)	1 (2.08%)
Death	4 (2.99%)	3 (3.49%)	1 (2.08%)

#### Dementia evaluation (MMST)

In total 17 patients (12.69%) had a MMST < 18, corresponding to advanced or even severe dementia (operative group: 5 [10.42%]; non-operative group: 12 [13.95%]).

#### Pain evaluation (NRS/PAINAD)

**Fig. 4:**
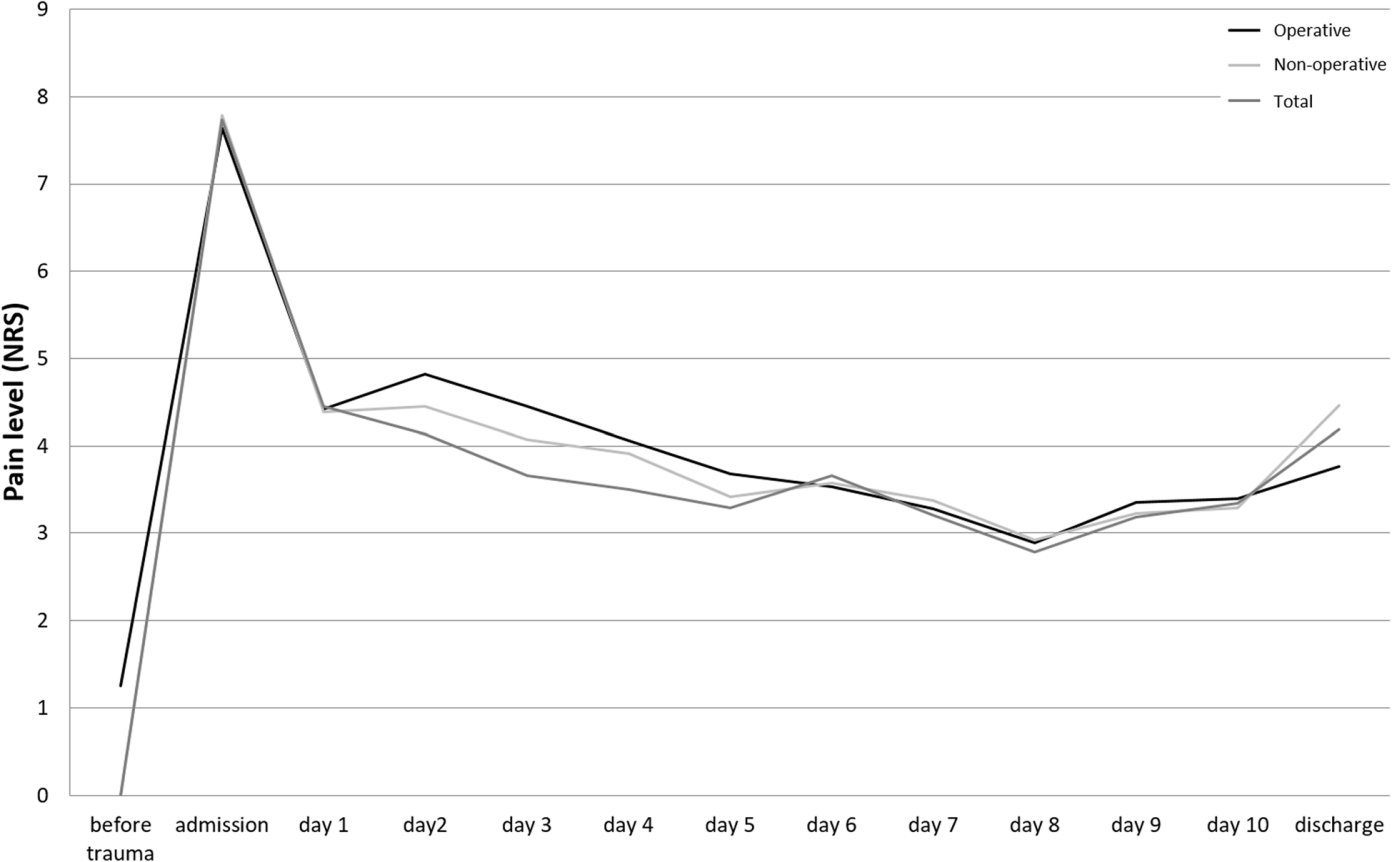
Pain level (NRS) before trauma, at admission, during hospital stay and at discharge

At the time of admission, NRS/PAINAD was 7.74 (±1.94) in total. At discharge, the average pain level (NRS/PAINAD) was 4.19 (±2.29). The difference was significant (*p* <  0.001). Data on pain level during all stages of hospitalization are shown in Fig. 4.

#### Non-operative group

NRS/PAINAD was 7.84 (±1.8) at admission and 5.59 (±2.2) at discharge in the non-operatively treated group. The difference was significant (*p* <  0.001).

#### Operative group

NRS/PAINAD was 7.57 (±2.1) at admission and 3.77 (±2.4) at discharge in the operatively treated group. The difference was significant (*p* <  0.001).

Statistically significant differences between the two groups regarding NRS/PAINAD were found on day three and day four. Whereas non-operatively treated patients showed a pain level of 3.26 (±2.21) on day three and 3.19 (±2.22) on day four, patients who underwent surgery had a pain level of 4.69 (±2.6) on day three and 4.83 (±2.17) on day four. Patients who had surgery before day four were excluded from this data analysis. On both days differences were statistically significant (*p* = 0.032 and *p* = 0.023 on day three and day four, respectively) (see Table 1).

#### Mobility admission and discharge

A significant difference was found in the mobility score between the score before trauma (13.43 (±2.7)) and at admission (2.09 (±3.89) (*p* <  0.001)). At discharge only 12 patients were fully mobilized (10.62%). Significant differences between the non-operative and operative groups were found in the mobility score before trauma and in the mobility score at admission (see Table 1). An additional significant difference was found in the mobility score regarding B2 fractures. The average mobility score difference between admission and discharge was higher in patients who suffered from B2 fractures and underwent surgery than in patients with B2 fractures and non-operative treatment (*p* = 0.035); see Table 4.

**Table 4:** Characteristics of the operative and conservative group (only type B2 fractures according to AO/ASIF) and differences between the groups. Values are presented in Mean, Standard Deviation (SD), Median and Interquartile Range (IQR); n = number of patients evaluated, if different from total number; NRS = Numeric Rating Scale, PAINAD = Pain Assessment in Advanced Dementia

**Only B2 fractures (AO/OTA)** (***n*** = 70)	Operative group (***n*** = 30) Mean (SD), *Median [IQR]*	Non-operative group (***n*** = 40) Mean (SD), *Median [IQR]*	***p***-value
Duration of hospital stay (d)	16.2 (±8,41)	8.59 (±5,44)	**< 0.001**
Local complications (patients)	4	0	**0.018**
Systemic complications (patients)	8	4	0.069
NRS/PAINAD at admission	7.57 (±2.11), *8 [3]*	7.78 (±1.93), *8 [2.25]*	0.468
NRS/PAINAD at discharge	3.44 (± 2.55), *3 [4.5]* (*n* = 25)	3.69 (± 2.06), *4 [3]* (*n* = 26)	0.542
Mobility before trauma	14.00 (± 2.75), *15 [0]*	13.25 (± 2.42), *15 [5]*	**0.07**
Mobility at admission	0.83 (± 3.24), *0 [0]*	1.88 (± 3.7), *0 [0]*	0.095
Standing possible (day)^a^	4.94 (±3.87), *4 [4.25]* (n = 14)	2.25 (±2.18), *2 [1.25]* (*n* = 32)	**0.003**
Walking in patient room possible (day)^a^	8.4 (±4.95), *6 [5.75]* (*n* = 10)	2.8 (±2.61), *2 [2]* (n = 25)	**< 0.001**
Mobility at discharge	6.29 (±4.72), *10 [10]* (n = 27)	4.53 (± 4.81), *5 [10]* (n = 30)	0.139
Mobility difference at admission/discharge	5.37 (± 5.71) (*n* = 27)	2.81 (± 4.39) (n = 32)	**0.035**

^a^Patients who were operated before day four were excluded from this examination

#### Mobility during hospitalization

**Fig. 5:**
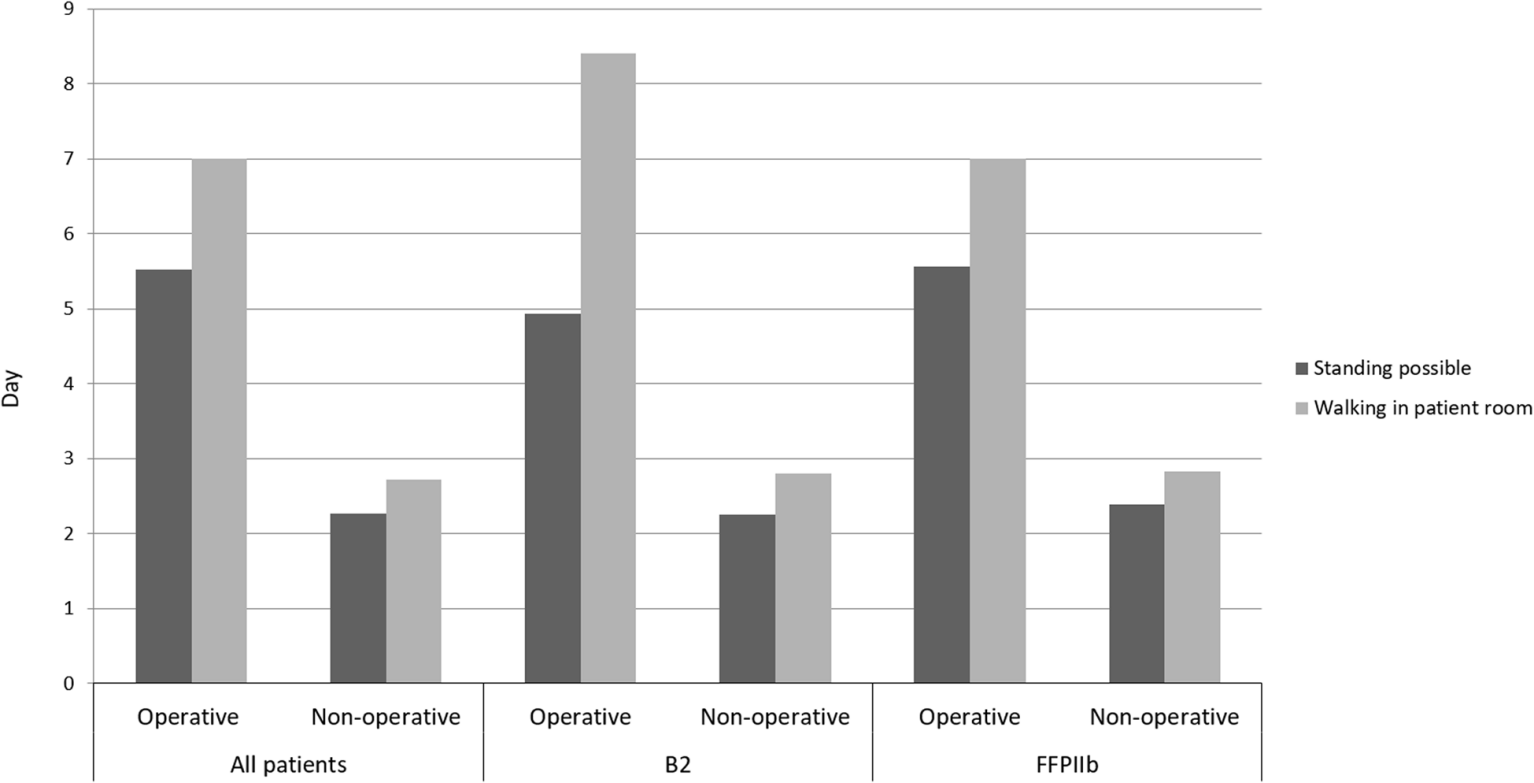
Level of mobilization during hospitalization (daily evaluation). To evaluate influence of mobility regarding operation decision, patients who were operated on before day four were excluded from this examination

To evaluate the influence of mobility regarding operation decision, patients who were operated on before day four were excluded from this examination. Statistically significant differences were found regarding the possibility to stand and the possibility to walk in the patient room (see Table 1 and Fig. 5). Regarding B2 and FFP IIb fractures, similar significant differences were detected (see Table 4 and Fig. 5).

## Discussion

Due to the increasing incidence of osteoporosis-associated pelvic fractures, clinically important challenges are arising regarding diagnostic evaluation, fracture classification and treatment algorithms [6]. Treatment goals should contain rapid pain relief and mobilization in order to avoid complications related to immobilization [6, 13]. Up to now there have only been retrospective evaluations regarding epidemiologic data, treatment options, complications and patient outcome. The aim of the present study was to describe specific characteristics of patients suffering from pelvic fragility fractures and evaluate factors that might influence treatment decisions which may optimize treatment pathways and patient mobility in the future.

Gender, age distribution as well as fracture type distribution in our study is comparable to that in the literature [2, 4, 7, 8, 19–21].

Several significant factors regarding surgical treatment decision were detected. First of all, fracture type significantly influenced treatment decision. Whereas A2 and FFP I fractures were treated non-operatively, fractures with high instability (FFP III, FFP IV, B3, C1 and C3) underwent in most cases a surgical procedure in accordance with current treatment recommendations [6, 13]. These findings are comparable to the retrospective evaluation by Höch et al. [22]. The average time of operation in our study was the fourth day after admission, whereas former studies report later times of operation [21, 23]. In our study B2 and FFP II fractures were treated non-operatively as well as operatively. For these fracture types, clear treatment recommendations are lacking, and classification systems (AO/OTA as well as FFP classification) are not useful for treatment decision regarding type B or FFP II fractures in the elderly. Operative treatment is recommended if pain level does not decrease via non-operative treatment [6, 13].

A significant reduction of the pain level during hospital stay was found in the non-operatively treated and in the surgically treated patient population. Significant differences between these groups regarding pain level were found on day three and four after admission. Patients who underwent a surgical procedure had a significantly higher pain level on day three and four compared to the non-operative group. Thus, pain level on day three and four was identified as an additional significant factor regarding treatment decision, leading to operations occurring on average on day four after admission. Pain level could be reduced significantly after operative treatment, comparable to the findings of Hopf et al., who showed significant reduction of pain level after stabilization of the posterior pelvic ring via iliosacral screw fixation [23].

Further significant differences were found regarding mobility levels. Patients treated with surgical procedures had a significantly higher mobility level before trauma as well as a significantly lower mobility level at the time of admission compared to patients treated with non-operative procedures. During hospitalization patients who underwent further non-operative treatment were able to stand in front of their bed and walk in the patient room on day three, whereas patients who were treated operatively on day four or later were not able to stand or walk on day three. Thus, mobility seems to be an additional factor influencing treatment decision.

In total, fragility fractures of the pelvic ring led to a significantly lower mobility level not only at the time of admission but also at the time of discharge from the hospital. Regarding B2 fractures a significantly higher mobility level difference between time of admission and time of hospital discharge was found in patients treated with surgical procedures compared to patients treated non-operatively. Comparable studies on mobility level are lacking, and these findings have to be discussed critically with respect to higher complication rates in the operatively treated population. Nevertheless, complication rates between operatively and non-operatively treated patients regarding B2 fractures showed no significant differences. Additionally, Höch et al. were able to show a higher two-year survival rate in patients who underwent surgery for B2 and B3 fractures [24].

**Fig. 6:**
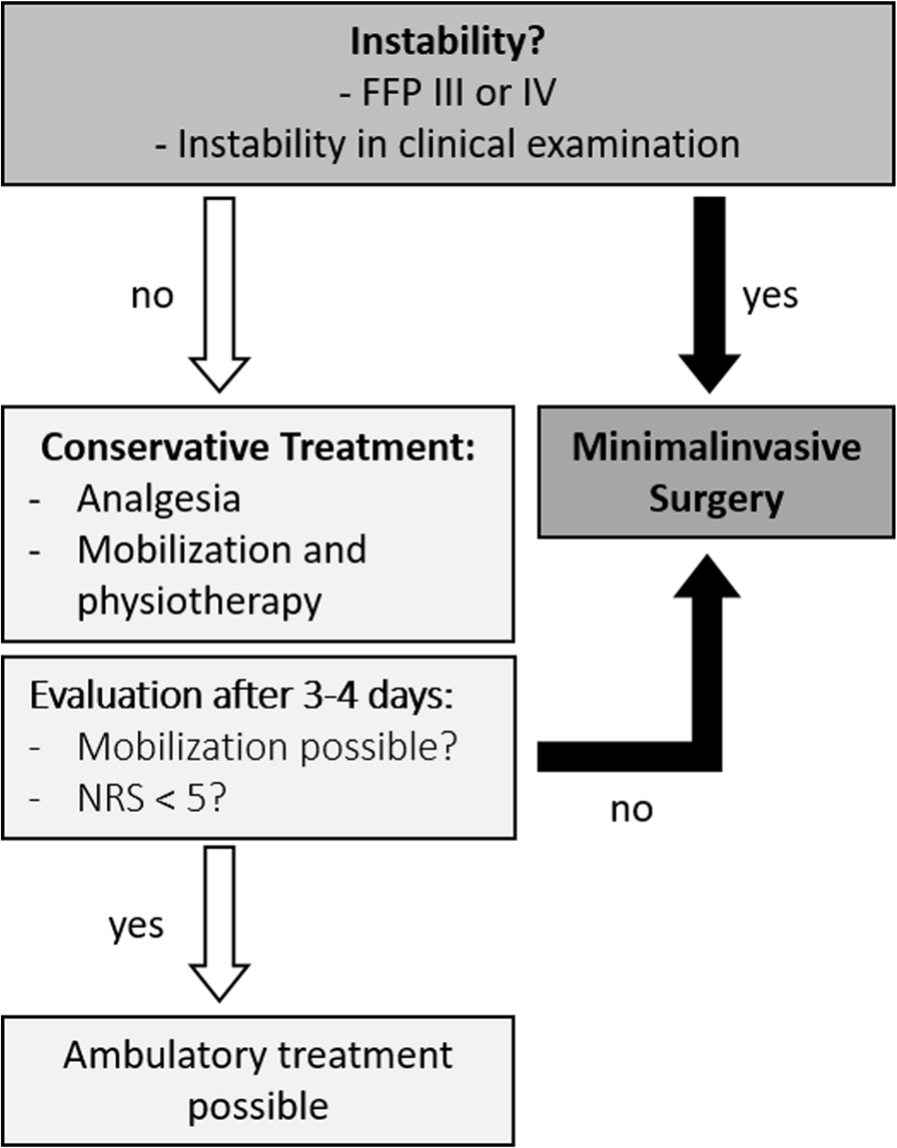
Proposed treatment algorithm of patients with fragility fractures of the pelvis (≥ FFP II). This treatment algorithm should only be considered as a suggestion and cannot be validated by the available data

Based on our findings we suggest a treatment algorithm, which is shown in Fig. 6. This treatment algorithm should only be considered as a suggestion and cannot be validated by the available data.

Nonetheless, our study has some limitations. Although the study design was prospective, the subgroups have a small sample size and have to be discussed critically. Furthermore only patients who were admitted to hospital were included in the study. This might result in a selection bias of the cohort. Mobility level was solely measured using a subsection of the Barthel index, and functional scores are lacking. Fractures were classified via CT diagnostics; no MRI was performed. This might have caused a bias in fracture type classification since some posterior compression fractures might not be detected in CT. Seventeen patients suffered from advanced or severe dementia. This also may have led to a bias regarding pain evaluation. To minimize bias, we used the PAINAD scale analogous to the NRS in patients with MMST < 18 points. Furthermore, data were collected solely for the duration of the hospital stay; long-term follow-up is lacking at this point in time. Finally, it should be mentioned that missing data also occurred during the evaluation of the outcome parameters (e.g. four patients died during hospitalization).

Nevertheless, this is the very first prospective study focused on treatment decision regarding fragility fractures of the pelvic ring. Treatment decisions regarding fragility fractures of the pelvis should be based upon a combination of several points and need to be evaluated implicitly in further prospective studies with a long-term follow-up in order to establish validated treatment algorithms and achieve the best possible outcome.

## Conclusion

Fragility fractures of the pelvis lead to significant reduction of mobility levels and high pain levels. In this prospective study several factors were identified regarding treatment procedure. Fracture type and mobility before trauma seem to influence treatment decision. Furthermore immobility on day three as well as a high pain level on day three and day four after admission influence the decision to proceed with surgical treatment. Patients suffering from B2 fractures in particular benefitted in terms of mobility level at discharge when treated operatively.

## Data Availability

The datasets generated and analyzed during the current study are not publicly available due to limitations of ethical approval involving the patient data and anonymity but are available from the corresponding author on reasonable request.

## Abbreviations

AO/OTA: Association for osteosynthesis / orthopaedic trauma association
FFP: Fragility fractures of the pelvis
ISS: Injury severity score
NRS: Numeric rating scale
ASA: American society of anesthesiologists
CT: Computed tomography
MRI: Magnetic resonance imaging
MMST: Mini-mental status test
PAINAD: Pain assessment in advanced dementia

## References

[1] Sullivan MP, Baldwin KD, Donegan DJ, Mehta S, Ahn J (2014). Geriatric fractures about the hip: divergent patterns in the proximal femur, acetabulum, and pelvis. Orthopedics..

[2] Kannus P, Palvanen M, Niemi S, Parkkari J, Järvinen M (2000). Epidemiology of osteoporotic pelvic fractures in elderly people in Finland: sharp increase in 1970-1997 and alarming projections for the new millennium. Osteoporos Int.

[3] Kannus P, Parkkari J, Niemi S, Sievänen H (2015). Low-trauma pelvic fractures in elderly Finns in 1970–2013. Calcif Tissue Int.

[4] Andrich S, Haastert B, Neuhaus E, Neidert K, Arend W, Ohmann C, Grebe J, Vogt A, Jungbluth P, Rösler G, Windolf J, Icks A (2015). Epidemiology of pelvic fractures in Germany: considerably high incidence rates among older people. PLoS One.

[5] Nanninga GL, de Leur K, Panneman MJM, van der Elst M, Hartholt KA (2014). Increasing rates of pelvic fractures among older adults: the Netherlands, 1986-2011. Age Ageing.

[6] Oberkircher L, Ruchholtz S, Rommens PM, Hofmann A, Bücking B, Krüger A (2018). Osteoporotic pelvic fractures. Dtsch Arztebl Int.

[7] Fuchs T, Rottbeck U, Hofbauer V, Raschke M, Stange R (2011). Pelvic ring fractures in the elderly. Underestimated osteoporotic fracture. Unfallchirurg..

[8] Rollmann MF, Herath SC, Kirchhoff F, Braun BJ, Holstein JH, Pohlemann T, Menger MD, Histing T (2017). Pelvic ring fractures in the elderly now and then ??? A pelvic registry study. Arch Gerontol Geriatr.

[9] Breuil V, Roux CH, Testa J, Albert C, Chassang M, Brocq O, Euller-Ziegler L (2008). Outcome of osteoporotic pelvic fractures: an underestimated severity. Survey of 60 cases. Jt Bone Spine.

[10] Melton LJ, Chrischilles EA, Cooper C, Lane AW, Riggs BL (2005). How many women have osteoporosis?. J Bone Miner Res.

[11] Tile M (1988). Pelvic ring fractures: should they be fixed?. J Bone Joint Surg Br.

[12] Rommens PM, Hofmann A (2013). Comprehensive classification of fragility fractures of the pelvic ring: recommendations for surgical treatment. Injury..

[13] Rommens PM, Wagner D, Hofmann A (2017). Fragility fractures of the pelvis. J Bone Jt Surg Rev.

[14] Maier GS, Kolbow K, Lazovic D, Horas K, Roth KE, Seeger JB, Maus U (2016). Risk factors for pelvic insufficiency fractures and outcome after conservative therapy. Arch Gerontol Geriatr.

[15] Taillandier J, Langue F, Alemanni M, Taillandier-Heriche E (2003). Mortality and functional outcomes of pelvic insufficiency fractures in older patients. Jt Bone Spine..

[16] Mahoney FI, Barthel DW (1965). Barthel index. Md State Med J.

[17] Warden V, Hurley AC, Volicer L (2003). Development and psychometric evaluation of the pain assessment in advanced dementia (PAINAD) scale. J Am Med Dir Assoc.

[18] Hack J, Kranz Y, Knauf T, Bäumlein M, Malcherczyk D, Ruchholtz S, et al. Stability of internal versus external fixation in osteoporotic pelvic fractures – a biomechanical analysis. Injury. 2020;51(11):2460–4. 10.1016/j.injury.2020.08.017 32800315

[19] Rommens PM, Ossendorf C, Pairon P, Dietz SO, Wagner D, Hofmann A (2015). Clinical pathways for fragility fractures of the pelvic ring: personal experience and review of the literature. J Orthop Sci.

[20] Wagner D, Hofmann A, Kamer L, Sawaguchi T, Richards RG, Noser H, Gruszka D, Rommens PM (2018). Fragility fractures of the sacrum occur in elderly patients with severe loss of sacral bone mass. Arch Orthop Trauma Surg.

[21] Höch A, Pieroh P, Henkelmann R, Josten C, Böhme J (2017). In-screw polymethylmethacrylate-augmented sacroiliac screw for the treatment of fragility fractures of the pelvis: a prospective, observational study with 1-year follow-up. BMC Surg.

[22] Höch A, Pieroh P, Gras F, Hohmann T, Märdian S, Holmenschlager F (2019). Age and “general health”—beside fracture classification—affect the therapeutic decision for geriatric pelvic ring fractures: a German pelvic injury register study. Int Orthop.

[23] Hopf JC, Krieglstein CF, Müller LP, Koslowsky TC (2015). Percutaneous iliosacral screw fixation after osteoporotic posterior ring fractures of the pelvis reduces pain significantly in elderly patients. Injury..

[24] Höch A, Özkurtul O, Pieroh P, Josten C, Böhme J (2017). Outcome and 2-year survival rate in elderly patients with lateral compression fractures of the pelvis. Geriatr Orthop Surg Rehabil.

